# Analysis of factors influencing skin sensitivity test results of antivenom and its potential correlation with allergic reactions: a retrospective cohort study

**DOI:** 10.3389/falgy.2026.1774186

**Published:** 2026-05-19

**Authors:** Haoyu Wang, Weiwei Qian, Shuyun Xu

**Affiliations:** 1 Emergency Department of West China Hospital, Sichuan University, Chengdu, Sichuan, China; 2 Department of Emergency, Shangjinnanfu Hospital, West China Hospital, Sichuan University, Chengdu, Sichuan, China

**Keywords:** allergic reactions, antivenom, skin sensitivity test, snakebite, snakebites

## Abstract

**Background:**

Snakebite remains a persistent public health concern, whether skin sensitivity testing for antivenom should be performed remains a subject of debate. Moreover, no study to date has been conducted to investigate the risk factors associated with antivenom skin sensitivity testing.

**Objective:**

This study aimed to analyze the factors influencing a positive skin test for antivenom following snakebite and to investigate the correlation between the antivenom skin test results, assessing the correlation between antivenom skin sensitivity testing and allergic reactions, as well as the necessity of performing such testing prior to antivenom administration.

**Methods:**

A retrospective analysis was conducted on snakebite cases presenting to our hospital between June 2013 and May 2020. Univariate and multivariate logistic regression analyses were employed to identify potential factors influencing a positive skin test reaction to antivenom in snakebite cases.

**Results:**

Among 2,495 patients with snakebite, the incidence of a positive skin test reaction to antivenom was 9.80%. The incidence of a positive skin test was significantly higher in female patients compared to male patients (11.56% vs. 8.32%, *P* < 0.05). Respiratory rate, thrombin time, and serum phosphorus level were identified as independent risk factors for a positive antivenom skin test in patients with snakebite (*P* < 0.05). Following skin sensitivity testing, comprehensive evaluation, desensitization therapy, and prophylactic anti-allergy treatment, the overall incidence of antivenom-related adverse events (including allergic reactions and serum sickness) was less than 1.5%.

**Conclusions:**

The findings of this study indicate that respiratory rate, thrombin time, and serum phosphorus level are independent risk factors for a positive result on the antivenom skin sensitivity test. A positive antivenom skin test result may be associated with the occurrence of allergic reactions. A comprehensive evaluation incorporating a positive skin test result to guide antivenom desensitization and prophylactic anti-allergy treatment may help reduce the incidence of allergic reactions and serum sickness. Therefore, the antivenom skin sensitivity test may serve as one of the sequential measures in the management and treatment of snakebite cases.

## 1 Introduction

High-quality antivenom serves as the most effective treatment for preventing or reversing the envenoming effects of snakebites and is now widely used globally (1–4). Despite this, snakebite envenoming remains a significant public health threat worldwide. Conservative estimates indicate that approximately 5 million people are bitten by snakes annually, resulting in over 100,000 deaths and leaving several hundred thousand individuals with permanent disabilities (4, 5). This phenomenon is attributed to multiple factors, including delays in patients seeking medical care, challenges in identifying the snake species responsible, as well as the high cost, variable efficacy, and significant adverse reactions associated with antivenom therapy (6–13).

Like any biological product, the administration of antivenom can lead to allergic reactions and serum sickness (14–16). Inappropriate management of these reactions may precipitate anaphylactic shock and even death. To mitigate such adverse events, some antivenom manufacturers and national guidelines mandate a pre-administration skin sensitivity test (17–19). This practice is theoretically based on the presumed correlation between antivenom reactions and a positive skin test result (14, 20). In China, performing a skin test is a mandatory step prior to antivenom infusion. Implementing this test is considered to aid in preventing antivenom-related adverse reactions, facilitating personalized treatment for snakebite victims, and potentially reducing the need for additional hospitalization (21–23).

However, considerable debate exists regarding the necessity of pre-administration skin testing for antivenom. Some argue that the skin sensitivity test has limited predictive value for antivenom-related reactions and that its necessity should not be overemphasized (20). Proponents of this view posit that the skin test primarily detects IgE-mediated type I hypersensitivity. In contrast, the vast majority of antivenom reactions are believed to result from direct, dose-related complement activation rather than non-dose-related, IgE-mediated hypersensitivity. Moreover, no study to date has been conducted to investigate the risk factors associated with antivenom skin sensitivity testing. Therefore, this study hypothesizes that skin test results are closely associated with allergic reactions. By analyzing the risk factors for a positive skin test reaction to antivenom in snakebite patients, this research aims to investigate the correlation of these risk factors with both a positive skin test and the occurrence of allergic reactions, thereby providing evidence to inform the safe administration of antivenom and the prevention of complications.

## 2 Materials and methods

### 2.1 Research design and objective

This retrospective observational study aimed to investigate the factors influencing a positive skin test reaction to antivenom in snakebite patients and to examine the correlation between early antivenom skin test results and the occurrence of allergic reactions. The findings are intended to provide a preliminary assessment for the safe administration of antivenom and the prevention of hypersensitivity reactions and serum sickness.

### 2.2 Research population

Snakebite cases presenting to the Emergency Department of West China Hospital of Sichuan University between June 1, 2013 and May 31, 2020 were identified. Patient data were extracted from the hospital's Laboratory Information System (LIS) and Hospital Information System (HIS).

#### 2.2.1 Inclusion criteria

This study retrospectively included snakebite-related cases between June 2013 and May 2020, encompassing patients with direct bites, confirmed venom exposure, or high clinical suspicion thereof. Inclusion was contingent upon the completion of the medical process, availability of comprehensive clinical records in the HIS, and documented informed consent for antivenom use.

#### 2.2.2 Exclusion criteria

Cases were excluded for the following reasons: registration without subsequent evaluation/treatment, duplication of records, or insufficient clinical/laboratory data.

### 2.3 Data collection

#### 2.3.1 Case collection methods

Clinical data for all snakebite cases were retrieved from the Hospital Information System (HIS), the Laboratory Information System (LIS), and paper-based medical records of West China Hospital of Sichuan University.

#### 2.3.2 Record research indicators

- Collected the general information of patients: gender, age, from snakebite to accept medical treatment, state of consciousness, snakebite's site, the history of allergy, and basic diseases such as diabetes and hypertension, and so on;
- Collected the first vital signs measured at the pre-examination triage include temperature, heart rate, respiratory rate, systolic blood pressure, diastolic blood pressure, and peripheral oxygen saturation;
- Collected the results of the first blood test before skin test, such as blood routine results, blood biochemical results, blood coagulation routine results. It comes from the LIS system of West China Hospital of Sichuan University.

#### 2.3.3 Endpoint indicator

The skin test result was the primary outcome of this study. The outcome of the skin test was categorized as either negative or positive. According to the instructions for antivenom use, the skin test was needed, for the test, 0.1 mL of antivenom diluted at 1:20 was injected intradermally into the forearm. A positive skin test was defined as local redness or indurations >5 mm in circumference at the injection site or any systemic allergic reactions within 30 min. In addition, adverse events after antivenom injection, which include anaphylaxis and serum sickness, were defined as study endpoints.

### 2.4 Quality control

To prevent adverse outcomes, all snakebite cases received prioritized diagnosis and treatment by a physician upon arrival at the emergency department. All blood samples were collected prior to the administration of the skin test. The skin tests were administered and their results interpreted by two experienced emergency department nurses. Data collection for this study strictly adhered to the predefined inclusion and exclusion criteria.

If the antivenom skin test is positive, it is imperative to perform a desensitization protocol and have an experienced physician evaluate the need for prophylactic anti-allergy treatment.

### 2.5 Statistical analysis

For continuous variables, normality was assessed using the Shapiro–Wilk test. Data conforming to a normal distribution are presented as the Mean ± Standard Deviation (Mean ± SD) and were compared between groups using the independent samples *t*-test. Non-normally distributed data are presented as the median (interquartile range) and were compared using the Mann–Whitney *U* test. Categorical data are expressed as frequency (percentage), and between-group differences were assessed using the Chi-square test. Univariate logistic regression analysis was employed to identify potential risk factors influencing hypersensitivity reactions to antivenom. Variables demonstrating statistical significance in the univariate analysis were subsequently entered into a multivariate logistic regression model to determine independent risk factors. All statistical analyses were performed using SPSS software (version 23.0) and Origin 2025 software. A two-sided *P*-value of less than 0.05 was considered statistically significant.

## 3 Results

**Figure 1:**
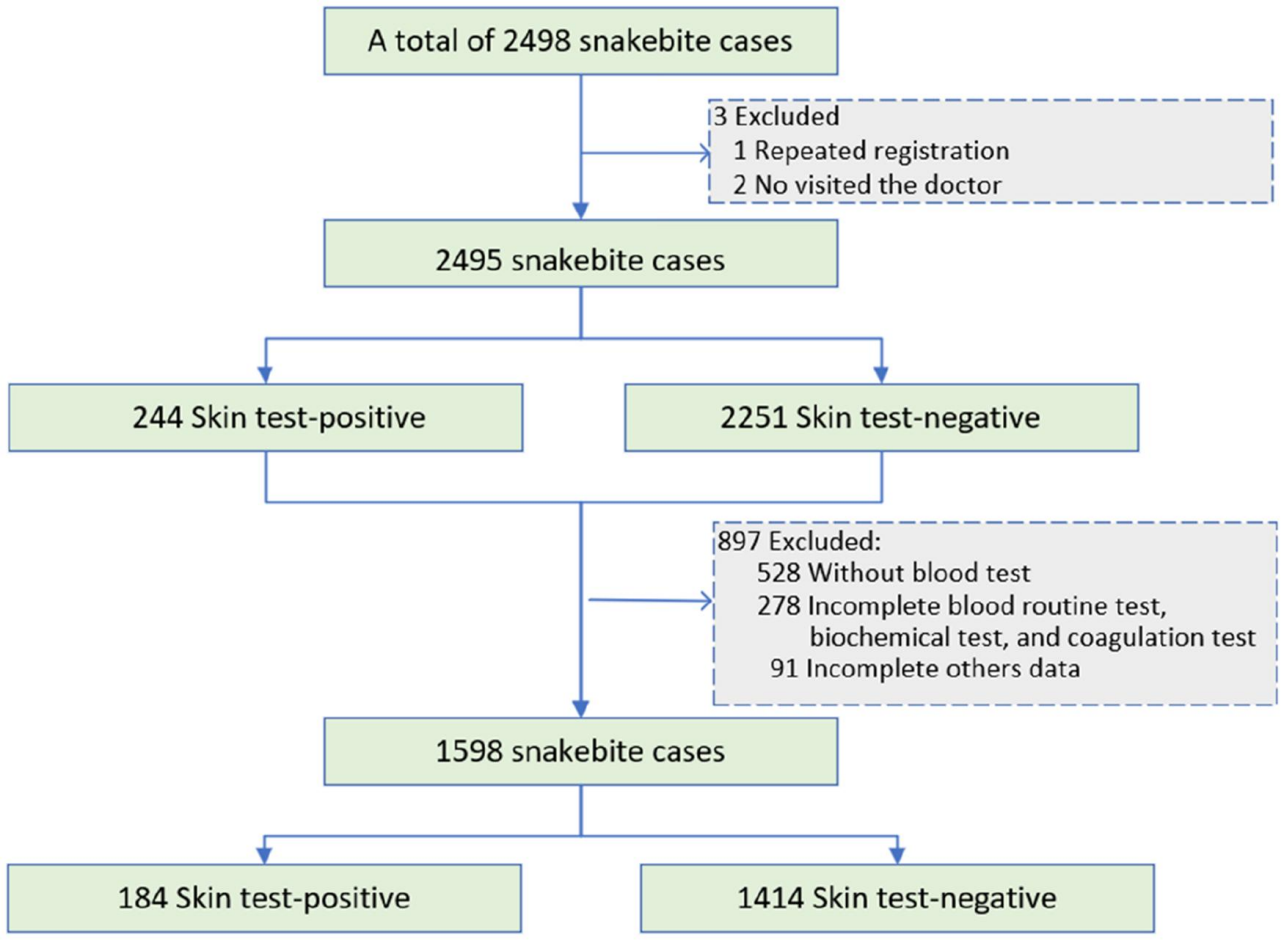
The detailed process of data extraction.

A total of 2,498 patients with snakebite presented to the emergency department between June 1, 2013 and May 31, 2020. Among them, 2,495 patients with snakebite underwent the antivenom skin test, of which 244 (9.80%) exhibited a positive reaction. After excluding 897 patients with incomplete laboratory data or other missing information, a total of 1,598 patients with snakebite were ultimately included in the univariate and multivariate logistic regression analyses (Figure 1). Among these, there were 184 patients (11.51%) with a positive skin sensitivity test result. This study found that fewer than 40 adverse events, including anaphylaxis and serum sickness, were reported, with an anaphylaxis frequency of approximately 1.5%. Based on a comprehensive review of medical records and physician orders in these cases, allergic reactions and serum sickness attributable to antivenom may have accounted for no more than 50% of incidents, with hypersensitivity reactions directly caused by the snakebite envenomation itself being more common.

Based on the skin test results, all cases were categorized into either a skin test-positive group or a skin test-negative group. No statistically significant differences were observed between the two groups regarding age, disease course, bite site, or history of allergy (*P* > 0.05) (Table 1). However, female patients demonstrated a significantly higher incidence of a positive skin test result compared to male patients (*P* < 0.05), with a positive rate of 11.56%.

**Table 1:** The comparison of patient's demographic data in patients.

Variables	Skin test-positive group (*n* = 244)	Skin test-negative group (*n* = 2,251)	*t*/*χ*^2^	*P*
Age	50.16 ± 18.347	50.80 ± 18.109	−0.525	0.885
Sex (%)				
Male	114 (46.72)	1,257 (55.84)	7.397	0.007
History of drug allergy (%)	7 (2.87)	45 (2.00)	0.816	0.344
Snakebite's site (%)				
Head	2 (0.82)	30 (1.33)	0.458	0.764
Upper limb	84 (34.42)	813 (36.11)	0.373	0.830
Lower limb	153 (62.70)	1,363 (60.55)	0.642	0.887
Body	2 (0.82)	10 (0.44)	0.648	0.331
Onset-to-door time (h)	14.25 ± 29.090	20.96 ± 62.456	−1.656	0.098

Normal distributed data were expressed as mean ± SD; enumeration data were expressed as frequency (ratio).

**Figure 2:**
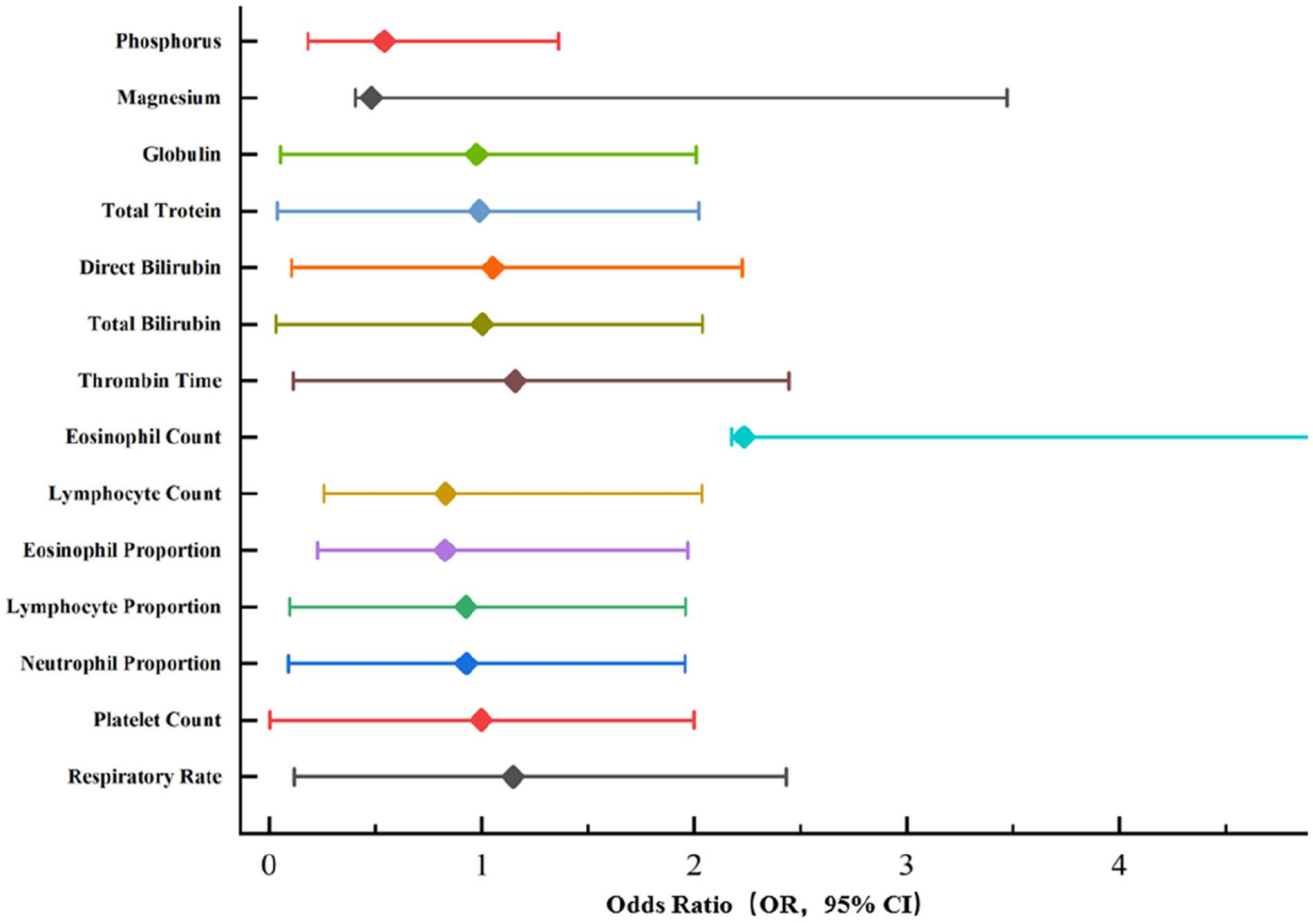
Forest plot of multivariable analysis on factors associated with positive skin test results following antivenin administration in snakebite patients. As the 95% CI (confidence interval) for the eosinophil count is exceptionally wide, the scale of this plot does not encompass its entire range.

The analysis of factors influencing a positive skin test reaction to antivenom included 1,598 snakebite cases (Table 2). The two groups showed no significant differences in age, sex, disease course, bite site, history of allergy, or underlying medical conditions (*P* > 0.05). Univariate logistic regression analysis identified the following factors as significant influencers of a positive skin test reaction (*P* < 0.05): respiratory rate, platelet count, percentage of segmented neutrophils, percentage of lymphocytes, percentage of eosinophils, absolute lymphocyte count, absolute eosinophil count, thrombin time, total bilirubin, direct bilirubin, indirect bilirubin, total protein, globulin, magnesium, and phosphorus (Table 3, Figure 2). Multivariate logistic regression analysis demonstrated that respiratory rate (Odds Ratio/OR 1.150, 95% Confidence Interval/CI: 1.030–1.285, *P* = 0.013), thrombin time (OR 1.159, 95% CI: 1.045–1.286, *P* = 0.005), and serum phosphorus level (OR 0.542, 95% CI: 0.359–0.820, *P* = 0.004) were independent risk factors for a positive skin test reaction to antivenom in snakebite cases (Table 3). Among these, respiratory rate was the only non-invasive independent risk factor identified. A negative correlation was observed between serum phosphorus level and a positive skin sensitivity test result.

**Table 2:** The comparison of patient's demographic data in patients.

Variables	Skin test-positive group (*n* = 184)	Skin test-negative group (*n* = 1,414)	*t*/*χ*^2^	*P*
Age	51.58 ± 17.896	52.08 ± 17.304	−0.369	0.712
Sex (%)				
Male	88 (47.82)	782 (55.30)	3.710	0.059
Snakebite's site (%)				
Head	2 (1.09)	23 (1.63)	0.308	0.760
Upper limb	60 (32.61)	501 (35.43)	0.569	0.461
Lower limb	121 (65.76)	887 (62.73)	0.642	0.465
Body	2 (1.09)	7 (0.50)	1.0198	0.278
Onset-to-door time (h)	14.73 ± 32.204	16.88 ± 47.085	−0.601	0.548
Past medical history (%)				
Hypertension	20 (10.87)	146 (10.32)	0.052	0.798
Diabetes	8 (4.35)	49 (3.47)	0.369	0.525
History of drug abuse	0 (0)	7 (0.50)	0.915	1.000
History of toxic	2 (1.09)	17 (1.20)	0.018	1.000
History of allergy	7 (3.80)	34 (2.40)	1.276	0.315

Normal distributed data were expressed as mean ± SD; enumeration data were expressed as frequency (ratio).

**Table 3:** The results of univariate and multivariate analysis for skin test positive after injected snake antivenom in snakebite patients.

	*P* *	OR	95% CI		*P* **	OR	95% CI	
			Lower	Upper			Lower	Upper
Basic information								
Age	0.712	1.002	0.993	1.011	-	-	-	-
Sex	0.055	0.740	0.544	1.006	-	-	-	-
Disease course (h)	0.550	1.001	0.997	1.005	-	-	-	-
Snakebite's site								
Head	0.582	1.505	0.352	6,435	-	-	-	-
Upper limb	0.451	1.065	0.904	1.254	-	-	-	-
Lower limb	0.423	0.957	0.859	1.066	-	-	-	-
Body	0.325	0.820	0.553	1.217	-	-	-	-
Vital sign								
Temperature	0.839	0.975	0.766	1.242	-	-	-	-
Heart rate	0.782	1.001	0.994	1.008	-	-	-	-
Respiratory rate	0.021	1.117	1.017	1.228	0.013	1.150	1.030	1.285
Systolic blood pressure	0.327	1.003	0.997	1.010	-	-	-	-
Diastolic blood pressure	0.157	1.004	0.998	1.010	-	-	-	-
Blood routine test								
Red blood cell count	0.965	1.006	0.786	1.287	-	-	-	-
Hemoglobin	0.467	1.003	0.995	1.011	-	-	-	-
Hematocrit	0.334	4,400	0.217	89.069	-	-	-	-
MCV	0.137	1.016	0.995	1.037	-	-	-	-
MCH	0.222	1.035	0.979	1.094	-	-	-	-
MCHC	0.773	0.998	0.985	1.011	-	-	-	-
RDW-CV	0.434	1.061	0.914	1.232	-	-	-	-
RDW-SD	0.059	1.039	0.999	1.081	-	-	-	-
Platelet count	0.008	0.997	0.995	0.999	0.470	0.999	0.997	1.001
White blood cell count	0.183	1.025	0.988	1.063	-	-	-	-
Neutrophil proportion	0.002	1.021	1.008	1.034	0.153	0.930	0.842	1.027
Lymphocyte proportion	0.000	0.973	0.959	0.988	0.171	0.926	0.829	1.034
Monocyte proportion	0.850	1.008	0.929	1.094	-	-	-	-
Eosinophil proportion	0.009	0.901	0.833	0.975	0.249	0.828	0.600	1.142
Basinophil proportion	0.300	0.577	0.204	1.632	-	-	-	-
Neutrophil count	0.070	1.035	0.997	1.075	-	-	-	-
Lymphocyte count	0.004	0.769	0.643	0.921	0.329	0.831	0.573	1.206
Monocyte count	0.417	1.292	0.696	2.401	-	-	-	-
Eosinophil count	0.016	0.292	0.108	0.794	0.649	2.235	0.061	88.231
Basinophil count	0.929	0.547	0.000	3,32,553.659	-	-	-	-
Coagulation function test								
Prothrombin time	0.705	1.007	0.973	1.041	-	-	-	-
INR	0.747	1.091	0.643	1.852	-	-	-	-
APTT	0.604	0.997	0.985	1.009	-	-	-	-
Thrombin time	0.004	1.153	1.045	1.272	0.005	1.159	1.045	1.286
Fibrinogen	0.365	0.918	0.762	1.105	-	-	-	-
Biochemical test								
Total bilirubin	0.006	1.027	1.008	1.047	0.880	1.003	0.970	1.036
Direct bilirubin	0.001	1.096	1.036	1.160	0.329	1.054	0.948	1.171
Indirect bilirubin	0.020	1.032	1.005	1.059	-	-	-	-
Total protein	0.007	0.967	0.943	0.991	0.594	0.989	0.950	1.030
Albumin	0.143	0.974	0.940	1.009	-	-	-	-
Globulin	0.005	0.950	0.916	0.985	0.392	0.975	0.922	1.033
Albumin/Globulin ratio	0.193	1.463	0.825	2.592	-	-	-	-
ALT	0.384	1.003	0.996	1.010	-	-	-	-
AST	0.141	1.003	0.999	1.008	-	-	-	-
AST/ALT ratio	0.102	1.086	0.967	1.454	-	-	-	-
Creatinine	0.338	1.003	0.997	1.009	-	-	-	-
Blood urea nitrogen	0.691	0.989	0.938	1.043	-	-	-	-
Uric acid	0.052	0.998	0.997	1.000	-	-	-	-
Glucose	0.163	1.046	0.982	1.114	-	-	-	-
Alkaline phosphatase	0.094	0.997	0.994	1.000	-	-	-	-
GGT	0.602	1.001	0.998	1.004	-	-	-	-
Calcium	0.275	0.580	0.218	1.542	-	-	-	-
Magnesium	0.029	0.160	0.031	0.827	0.433	0.482	0.078	2.989
Phosphorus	0.003	0.574	0.400	0.825	0.004	0.542	0.359	0.820
Sodium	0.911	1.003	0.957	1.051	-	-	-	-
Potassium	0.686	1.082	0.739	1.583	-	-	-	-
Chloride	0.675	0.991	0.951	1.033	-	-	-	-
CO_2_CP	0.568	1.014	0.967	1.063	-	-	-	-
Anion gap	0.957	1.001	0.957	1.048	-	-	-	-
Cystatin C	0.542	1.186	0.685	2.053	-	-	-	-
β-hydroxybutyric acid	0.398	0.824	0.526	1.291	-	-	-	-

MCV, mean corpuscular volume; MCH, mean corpuscular hemoglobin; MCHC, mean corpuscular hemoglobin concentration; RDW-CV, red cell distribution width CV; RDW-SD, red cell distribution width SD; INR, international normalized ratio; APTT, activated partial thromboplastin time; ALT, alanine amino transferase; AST, aspartate amino transferase; GGT, glutamyl gamma-glutamyl transpeptidase; CO_2_CP, carbon dioxide combining power.

* *P*: *P*-value from univariate analysis.

** *P*: *P*-value from multivariate logistic regression analysis.

**Figure 3:**
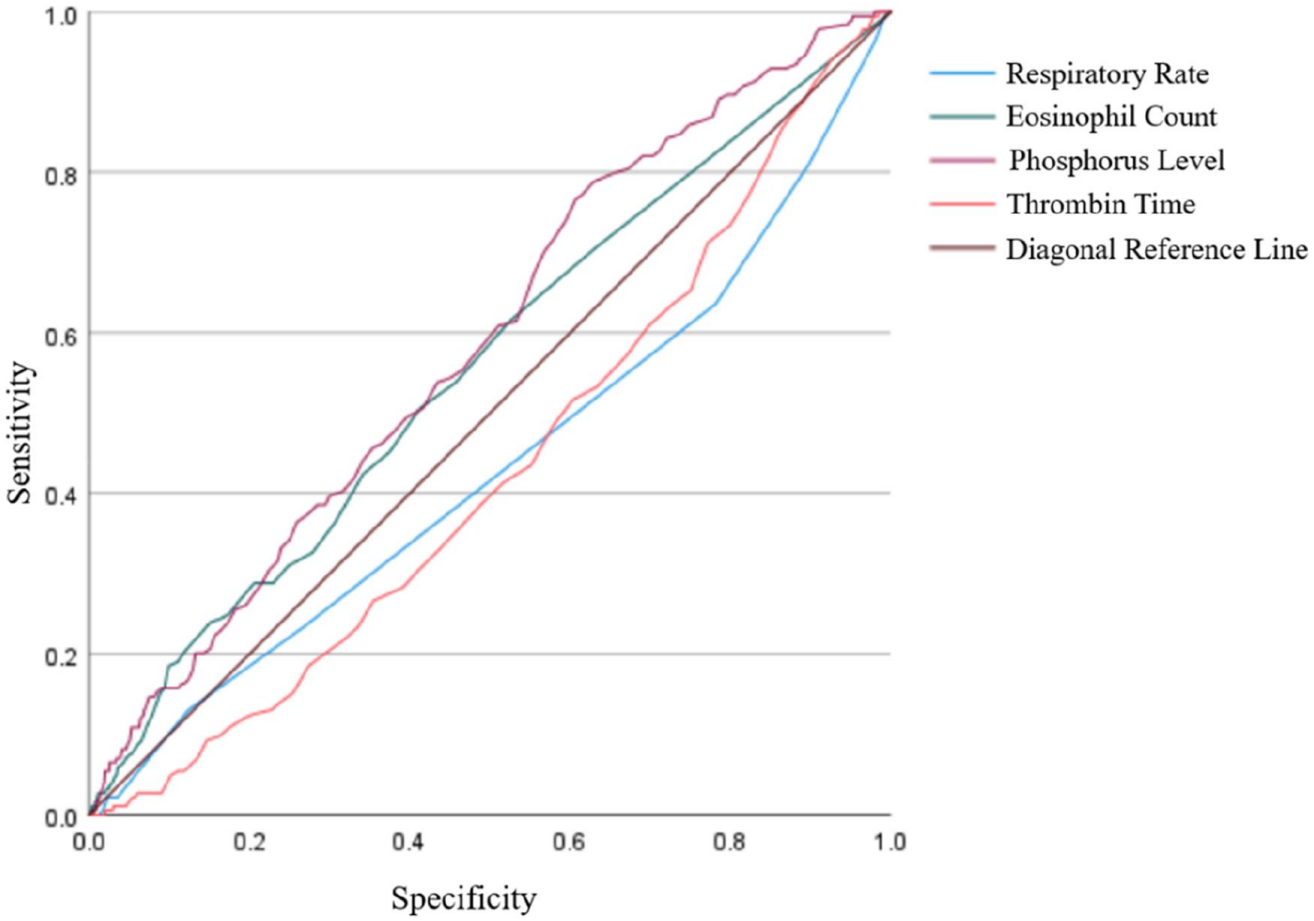
Comparison of ROC curves for different predictors.

Further Receiver Operating Characteristic (ROC) curve analysis revealed that serum phosphorus level (AUC 0.586, 95% CI 0.544–0.628) had the highest predictive value for a positive antivenom skin test, while the AUCs for respiratory rate, thrombin time, and eosinophil count were 0.433 (95% CI 0.386–0.480), 0.431 (95% CI 0.389–0.473), and 0.558 (95% CI 0.513–0.602), respectively (Figure 3).

Additionally, considering the geographical context of this study, local snake species distribution, and the pathophysiology of envenomation, we sought to investigate whether overall coagulation abnormalities influence the antivenom skin test result. Based on coagulation profiles, the cohort was stratified into a coagulation dysfunction group and a normal coagulation group. Coagulation dysfunction was defined as meeting at least one of the following criteria: prolonged prothrombin time, prolonged activated partial thromboplastin time, or prolonged thrombin time. The results indicated that 222 cases (13.89%) exhibited coagulation dysfunction. Within this subgroup, 21 cases (9.46%) had a positive skin test, compared to 163 positive cases (11.84%) in the normal coagulation group. This difference was not statistically significant (*P* = 0.311).

## 4 Discussion

This work represents the first investigation into the risk factors contributing to a positive antivenom skin test, with the identification of several predictive factors. This study found that the overall incidence of a positive skin test reaction to antivenom in snakebite cases was approximately 10%. Female patients demonstrated a significantly higher propensity for a positive skin test result (*P* < 0.05). It is well-established that gender is a significant factor influencing the incidence of various diseases, particularly allergic conditions (24–26). Studies have indicated that females are more susceptible to adverse reactions following vaccination, a phenomenon potentially linked to sex hormone levels (27). This finding suggests a potential correlation between antivenom skin test results and hypersensitivity reactions.

Our analysis identified respiratory rate as an independent risk factor for a positive skin test. Alterations in respiratory rate represent a common clinical manifestation of snakebite envenomation, influenced by numerous factors including stress, psychological impact, pain, direct venom toxicity, and allergic reactions (28). This result underscores the potential value of respiratory rate as a crucial parameter in the initial assessment of snakebite victims. However, the possibility of bradypnea must also be considered, as envenomation by certain species, such as deinagkistrodon acutus, trimeresurus stejnegeri, and sea snakes, can induce respiratory paralysis and fatalities (29, 30). In the region covered by this study, the predominant snake species is *Agkistrodon halys*; therefore, decreased respiratory rate was rarely observed. We believe that further focused studies are needed to confirm this finding.

Furthermore, thrombin time was established as an independent risk factor for a positive skin test. To mitigate the potential confounding effect of systemic coagulopathy on the skin test outcome, we specifically investigated its influence. The results indicated no statistically significant association between overall coagulation dysfunction, as defined by our criteria, and the skin test result (*P* > 0.05). Coagulopathy is a severe complication of snakebite, and antivenom remains the only specific antidote for its management (31, 32). Consequently, coagulation profiling is widely recommended for envenomated patients (22, 33). Some evidence suggests that the severity of coagulation abnormalities correlates with the venom load (34). A higher venom load implies greater introduction of allergens into the systemic circulation, potentially increasing the risk of hypersensitivity reactions in snakebite victims. In Southwest China, pit vipers (family Viperidae) are predominant, whose venoms primarily exert hematotoxic effects. The activation of the coagulation cascade can lead to consumptive coagulopathy, potentially triggering severe complications such as disseminated intravascular coagulation, hypovolemia, hypotension, and shock. Given that our study focused on the early phase of snakebite, thrombin time may serve as one of the indicators for predicting early hypersensitivity reactions.

This study identified serum phosphorus level as an independent risk factor for a positive skin test reaction to antivenom. A negative correlation was observed between serum phosphorus level and a positive skin sensitivity test result. Trace elements are essential components of the human body and play vital roles. Existing research suggests a potential link between trace elements and allergic diseases (35). Specifically, serum phosphorus levels have been reported to be inversely correlated with allergic conditions, where lower levels may increase the risk of hypersensitivity (36). Our findings align with this, demonstrating that snakebite patients with lower serum phosphorus levels were more likely to exhibit a positive skin test. This consistency further supports the potential correlation between antivenom skin test results and allergic susceptibility.

We advocate for the mandatory completion of skin sensitivity testing prior to antivenom administration. Although a positive skin test does not unequivocally predict an allergic reaction, it demonstrates a significant association. The highest odds ratio (OR 2.235) observed in the multivariate analysis for the absolute eosinophil count substantially reinforces this conclusion. We observed that some patients had already exhibited local or systemic allergic reactions prior to receiving antivenom, which was one of the reasons we did not further analyze risk factors for allergic reactions. We recommend that in future studies, when assessing allergic reactions or serum sickness associated with antivenom, careful differentiation should be made as to whether these reactions are caused by snake venom or antivenom. While the overall incidence of a positive skin test in our cohort was approximately 10%, the actual incidence of adverse reactions to antivenom during treatment was considerably lower than globally reported rates. This can likely be attributed to the detailed evaluation and prophylactic interventions implemented based on skin test results, particularly for positive cases. These measures included desensitization protocols, administration of antihistamines, and corticosteroids.

However, our further ROC analysis revealed that serum phosphorus level, respiratory rate, eosinophil count, and thrombin time had limited predictive efficacy for a positive antivenom skin test, with AUC values of 0.586, 0.433, 0.558, and 0.431, respectively. Despite reaching statistical significance in regression analysis, none of the identified independent risk factors demonstrate clinically meaningful predictive performance in isolation. This suggests that there is currently no reliable single predictor, indicating that positive skin tests and allergic reactions to antivenom in patients with snakebite result from multifactorial interactions. Therefore, prophylactic skin testing remains irreplaceable in clinical practice, and future assessment should be based on integrated multiparameter models.

The World Health Organization has set an ambitious target to halve the global burden of death and disability from snakebite by 2030 (4). Achieving this goal hinges on several critical factors: the early administration of antivenom, prompt in-hospital recognition and prevention of snakebite complications, and the mitigation of antivenom-related adverse reactions (37). First, it is imperative to produce sufficient quantities of high-quality, affordable antivenom (38). This is crucial for enhancing its effectiveness and minimizing adverse effects. Second, addressing the inequitable distribution of antivenoms and increasing the stockpiles of region-specific antivenoms is essential (39, 40). Finally, continued efforts to improve the early management skills of clinicians, enabling timely identification and prevention of adverse reactions, are paramount (41–44).

This study possesses significant novelty. It represents the first investigation into the risk factors for a positive antivenom skin sensitivity test and explores its correlation with allergic reactions. The findings suggest a positive association between a positive skin test result and the occurrence of allergic reactions. Therefore, a comprehensive assessment integrating skin test results to guide prophylactic anti-allergy treatment may help reduce the incidence of both allergic reactions and serum sickness. However, this study has several limitations. First, as a retrospective single-center study, its findings may primarily reflect regional characteristics and lack broad generalizability. Second, Owing to the limited number of adverse events, particularly anaphylaxis and serum sickness, following antivenom administration in this study, the statistical power was insufficient to further evaluate the risk factors. This was due to the preliminary data statistics indicating an exceedingly low number of such adverse events (fewer than 40 cases) over the 7-year study period, resulting in an incidence rate of less than 1.5%. Finally, the potential influence of unmeasured confounding factors cannot be excluded. Therefore, further research is warranted to validate these findings.

## 5 Conclusion

In our center, the overall incidence of a positive skin test reaction to antivenom in snakebite patients was approximately 10%, respiratory rate, thrombin time, and serum phosphorus level were identified as independent risk factors for a positive skin test. A positive correlation was observed between respiratory rate and thrombin time with a positive skin test result, whereas serum phosphorus level demonstrated a negative correlation. Based on our findings, a positive antivenom skin test result may be highly correlated with the occurrence of allergic reactions and can guide the evaluation of clinical treatment strategies to reduce the incidence of adverse effects. A comprehensive evaluation incorporating a positive skin test result to guide antivenom desensitization and prophylactic anti-allergy treatment may help reduce the incidence of allergic reactions and serum sickness. Therefore, the antivenom skin sensitivity test may represent one of the sequential measures in the management and treatment of snakebite cases.

## Data Availability

The raw data supporting the conclusions of this article will be made available by the authors, without undue reservation.
